# Individualized controlled ovarian stimulation in expected poor-responders: an update

**DOI:** 10.1186/s12958-018-0342-1

**Published:** 2018-03-09

**Authors:** Thor Haahr, Sandro C. Esteves, Peter Humaidan

**Affiliations:** 1 Department of Clinical Medicine, Aarhus University, Denmark and the Fertility Clinic Skive, Skive Regional Hospital, Skive, Denmark; 2 ANDROFERT, Andrology and Human Reproduction Clinic, Campinas, Brazil; 3 Department of Surgery (Division of Urology), Faculty of Medical Sciences, University of Campinas (UNICAMP), Campinas, Brazil

**Keywords:** Poor ovarian response, Bologna criteria, Poseidon classification, ART calculator, Controlled ovarian stimulation, Adjuvant therapy

## Abstract

Controlled ovarian stimulation with subsequent multi-follicular development continues to be a keystone in ART. Evidence supports an individualized approach to ovarian stimulation, usually involving combinations of ovarian reserve tests, body mass index and age to tailor the exogenous gonadotropin dose, and potentially adjuvant treatment aiming for high safety and a shortening of time to live birth. While stimulation and trigger concepts have been developed successfully in normo- and hyperresponder patients, the poor responder patient remains difficult to manage. However, recent advances in definition and classification of the expected poor ovarian responder patient might enable a more accurate and clinically useful interpretation of new treatment concepts in a more homogenous study population. In the present review, we discuss the classification of the expected poor ovarian responder patient as well as clinically useful measurements of efficacy for controlled ovarian stimulation, and finally, we discuss the evidence for clinical management of patients with expected poor ovarian response, including adjuvant treatments such as growth hormone, androgens, and LH activity.

In conclusion, the best available evidence supports that the treatment of the expected poor ovarian response patient should be individualized in all steps of ART, including the choice of GnRH analogue, the gonadotropin type and dose, ovulation trigger, and the possible use of adjuvant therapies.

## Background

In modern assisted reproductive technology (ART), as in any medical specialty, individualized treatment is the optimal goal to counterbalance efficacy and safety with the implementation of different ART modalities, optimizing time to live birth. In general, patients embarking on ART treatment can be reassured that they have a relatively good prognosis of obtaining a live birth. Based on a total of 5165 patients from the ART registry in Denmark during the period 2007–2010, a recent study reported a cumulative live birth rate of 71% (95% CI; 70–72%) at 5-year follow-up from the start of treatment [1]. Women aged < 35 years had an 80% live birth rate compared to 61% for women aged 35–39 years and 26% for women aged ≥40 years, emphasizing the importance of giving ART patients an age-stratified prognosis during counseling. With the exponential growth in technological advances, controlled ovarian stimulation (COS) remains the keystone of successful ART treatment, aiming at achieving multi-follicular development to obtain a good chance of transferring embryos with the highest implantation potential [2]. In agreement with recent reports comparing individualized and conventional COS [3, 4], most clinicians use ovarian reserve markers like antral follicle count (AFC) and/or Anti-Müllerian hormone (AMH) for clinical decision-making to tailor the most optimal individualized controlled ovarian stimulation (iCOS) strategy, securing the shortest time to pregnancy and live birth as well as a low risk of ovarian hyperstimulation syndrome (OHSS) development [5]. Thus, efficacy, safety and patient friendliness have become the mantras of modern ART, introducing protocols which decrease OHSS to an absolute minimum without compromising live birth rates [6, 7]. However, the clinical management of patients with poor ovarian reserve, so called poor ovarian responders (PORs) still remains a clinical challenge. This was further complicated by the fact that only until recently there was no general agreement about the diagnosis of POR. Thus, Polyzos and Devroey (2011) reported the use of as many as 41 different POR definitions in a total of 47 randomized controlled trials (RCT), which hampered the clinical value of interstudy comparison and meta-analysis in this heterogenous group of patients [8]. In their title the authors provocatively asked whether there was any “light at the end of the tunnel for the POR patient”. Subsequently, in 2011, an ESHRE consensus group [9] took the effort to try to standardize the definition of POR, establishing the so called ESHRE Bologna criteria (Table 1).

**Table 1:** The ESHRE consensus Bologna criteria for poor ovarian response (POR)

Advanced maternal age (≥40 years) or any other risk factor for POR^a^
A previous POR (≤3 oocytes with a conventional stimulation protocol)^b^
An abnormal ovarian reserve test (i.e. antral follicle count < 5–7 follicles or AMH < 0.7–1.3 ng/mL)^a,c^

Two out of three criteria need to be fulfilled in order to be defined as POR

^a^Expected poor responder if age ≥ 40 years and abnormal ovarian reserve test

^b^Poor responder if two previous episodes of POR after maximal stimulation

^c^AMH values updated in 2014, originally 0.5–1.1 ng/mL

Now, 6 years later, we ask ourselves, whether the Bologna criteria really brought POR patients out of the tunnel and into the light? In this review, we discuss the recent advances in iCOS for POR patients following the introduction of the ESHRE Bologna criteria. Moreover, we introduce the new POSEIDON classification of the “low prognosis patient” [10, 11], which was established with the primary objective of providing a more detailed stratification of expected low responders and to significantly reduce the heterogeneity seen in the Bologna POR population.

## Bologna criteria

The ESHRE Bologna criteria were primarily established to define the POR population based on strict criteria. The underlying idea was that this would secure future prospective RCTs of a more homogenous group when comparing new treatment modalities for POR patients [9]. Although the Bologna criteria was a crucial step towards defining POR, it became clear that even when using the Bologna criteria, the POR population remained heterogeneous primarily because the criteria did not adequately take the age-related impact on oocyte quality into consideration, which obviously significantly impacts success rates [12, 13]. Moreover, confusion existed between real poor ovarian response (poor ovarian reserve) and the cause of the poor response [14]. While yet to be proven in intervention trials based on the pharmacogenomic approach, a patient with an FSHR or LHR polymorphism [15], or the presence of variant LH *β* [16] with a good ovarian reserve could end up being classified as a Bologna POR patient because the ovarian stimulation was inadequately performed. Finally, in the Bologna criteria no recommendations for clinical decision-making were given.

Unfortunately, these aspects negatively impacts the design of RCTs because the clinical handling – and outcome would differ for different Bologna POR groups [13, 17, 18]. Regarding the age-related effect on oocyte quality, the Bologna criteria were criticized for the arbitrary cut-off value of 40 years of age [19], which based on ROC-curve analyses, age related aneuploidy rates, and the prognosis of live birth should optimally have been set at 35–37 years of age to better discriminate the POR population [1, 20–22]. Despite this criticism, an updated report in 2014 did not significantly change the Bologna criteria [23].

## Poseidon criteria

**Fig. 1:**
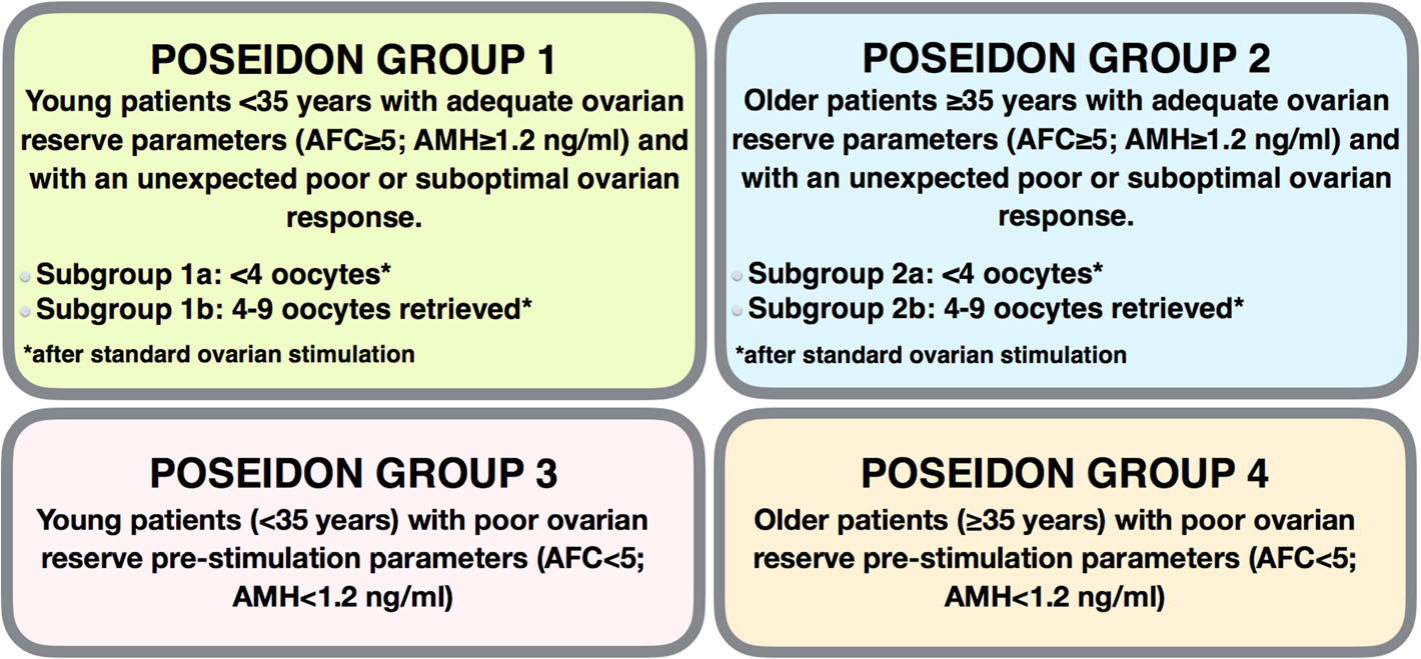
POSEIDON classification

In 2016 a group of reproductive endocrinologists and scientists gathered to further refine the definition of POR [11]. As a result, the new POSEIDON (**P**atient-**O**riented **S**trategies **E**ncompassing **I**ndividualize**d O**ocyte **N**umber) classification was developed, providing a more detailed classification to reduce the heterogeneity of the Bologna criteria. In brief, according to the POSEIDON classification patients are sub-divided into four sub-groups based on quantitative and qualitative parameters, namely: (i) age (ii) antral follicle count and/or AMH (iii) ovarian response – if a previous stimulation was performed (Fig. 1). Hence, the expected poor responder patient according to the age of the patient is classified as either Poseidon Group 3 or 4.

## One more oocyte matters

When considering the efficacy of iCOS, the introduction of the follicular output rate (FORT) [24] suggested a new measure of ovarian response to exogenous stimulation by assessing the ratio between the number of pre-ovulatory follicles obtained in response to gonadotropin administration and the pre-existing pool of small antral follicles. The FORT concept might even be taken a step further, including also the ratio between the final number of oocytes retrieved correlated to the antral follicle count (AFC) to measure successful ovarian response. As an example, a patient with a poor ovarian reserve who finally ends up with 70% of the antral follicles, resulting in retrieved oocytes has got a high FORT, and in reality, a good ovarian response to stimulation regardless of the total number of oocytes retrieved. In contrast, a patient with PCO like ovaries who ends up with less than 30% of her AFC resulting in retrieved oocytes should be considered as having a low FORT and a sub-optimal ovarian response, unless of course a suboptimal response was intended due to the risk of OHSS. In the majority of cases, a poor response in good reserve patients is caused by too low FSH dosing and the omission of LH activity supplementation in patients with the presence of LH β variant or LHR polymorphisms. Hence, FORT might be considered a quality marker of the iCOS strategy used [25]. During recent years it has been suggested that the retrieval of one more oocyte increases the predicted LBR per cycle [26]; i.e. retrieving three instead of two oocytes increases the predicted LBR by approximately 25%, relatively, in all age groups. Interestingly, this relative increase in LBR was also reported comparing PORs (< 5 oocytes) with suboptimal responders (5–9 oocytes) in a large retrospective study based on national registry data from 2005 to 2012 in Switzerland [27].

In this aspect one might ask, how many oocytes are actually needed to achieve the highest live birth rate (LBR) in the fresh cycle as well as after accumulation of the fresh and frozen transfer cycles deriving from one stimulation cycle (i.e. cumulative LBR). This question was initially analyzed by Drakopoulos et al. (2016) in a retrospective cohort of 1099 consecutive patients undergoing their first ovarian stimulation with a planned single embryo transfer [28]. The authors concluded that the low response patient with a mean age of 31 years (1–3 oocytes) had a significantly lower cumulative LBR (22%) compared to other sub-groups, including the suboptimal response patient (4–9 oocytes) in whom the cumulative LBR was 40% and the optimal response patient (10–15 oocytes) for whom the cumulative LBR was 51%. These findings were recently corroborated by Zhou et al. (2017) in patients aged 35–40 years [29]. Again, the low response patient (1–4 oocytes) had the lowest cumulative LBR (37%) as compared to other sub-groups.

For the ageing patient, the most obvious reason for the decline in the cumulative LBR is the decrease in not only oocyte quantity, but also quality in terms of aneuploidy. Thus, whereas in the young woman (≤ 35 years) the euploidy rate of blastocysts is approximately 60%, it is as low as 30% in women between the age of 40–42 years, decreasing to 15% in women older than 42 years [21].

New evidence from basic science studies provide biologically plausible explanations for the age-related effects on aneuploidy rates which seem to be driven by impaired mitochondrial function, increased granulosa cell apoptosis, and increased levels of oxidative stress in germline cells [30].

## The ART calculator: Estimating the number of oocytes needed to achieve at least one euploid blastocyst

As previously discussed, the continuous and inexorable age-related decrease in oocyte quantity and quality results in fewer euploid embryos for transfer. It is, therefore, anticipated that the expected POR patient (POSEIDON groups 3 and 4), particularly the ageing group (POSEIDON group 4) would achieve a lower live birth rate than younger counterparts with normal/high ovarian reserve [31].

The availability of at least one euploid embryo for transfer evidently changes the fate of the expected POR patient, as approximately 60% of euploid blastocysts implant across all age categories [32]. Indeed, the higher the number of oocytes retrieved, the higher the probability of obtaining an embryo cohort that may include at least one euploid blastocyst [15]. In practical terms, however, the retrieval of a large number of oocytes may not be feasible in the expected POR due to reduced ovarian reserve. Naturally, the question clinicians may ask is: what is the number of oocytes needed to achieve at least one euploid embryo in a given expected POR patient? This is an important issue as it represents a logical endpoint to guide clinicians develop an individualized treatment plan. In fact, the ‘ability to retrieve the number of oocytes needed to obtain at least one euploid embryo for transfer’ was proposed by the POSEIDON group as a new measure of success in ART [10, 11].

Clearly, the estimation mentioned above must take into account the critical variables, affecting the probability of achieving the desired outcome, including (i) the expected embryo euploidy rate per age group, (ii) blastulation rate, (iii) fertilization rate, (iv) and number of mature oocytes, as all of them are indispensable for calculating the number of oocytes required to achieve at least one euploid blastocyst. A rough calculation using the average of commonly reported laboratory key performance indicators (KPI) suggest that 4–7 oocytes are needed in a younger patient with expected POR, whereas at least 12 oocytes would be required in older counterparts. However, a meticulous evaluation of a Fertility Center’s database shows that fertilization and blastulation rates will be affected by sperm parameters such as source, and status (fresh or frozen-thawed) of gametes [33]. Along the same lines, embryo euploid rates vary not only by female age, but possibly also with other confounders [34], thus making such estimations complicated and labor intensive.

In this aspect, a pretreatment prediction model – the ART Calculator - using age and other predictors has been developed to estimate the number of oocytes required to achieve at least one euploid blastocyst for transfer after an IVF/ICSI treatment. The model was constructed based on the results of the Lasso (least absolute shrinkage and selection operator) regression analysis, which was utilized for both variable selection and regularization to enhance the prediction accuracy and interpretability of the statistical model. The ‘ART Calculator’ is available online at http://www.groupposeidon.com/ and is fully aligned with the POSEIDON marker of successful outcome.

## iCOS in expected poor responders (Poseidon groups 3 and 4)

**Fig. 2:**
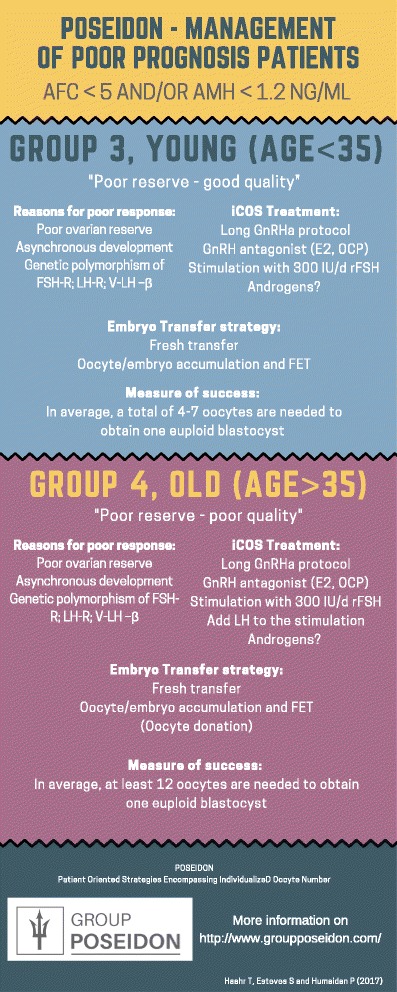
POSEIDON – management of poor prognosis patients

As seen from Fig. 2, POSEIDON categorizes the expected POR patient into two groups, stratifying according to age and using a cut-off of 35 years, a total AFC < 5, and/or an AMH < 1.2 ng/ml.

### GnRH analogue

When evaluating the evidence for clinical handling of the expected POR patient one has to distinguish between studies performed before and after the introduction of the Bologna criteria. Prior to the consensus as mentioned, multiple definitions of POR were used, introducing heterogeneity and subsequent poor clinical value of the reported results, in particular those of meta-analyses. As regards IVF/ICSI, Danhua et al. (2011) published a meta-analysis on non-Bologna criteria POR studies exploring the optimal GnRH analogue treatment. From this analysis, it was concluded that there was no statistically significant difference in clinical pregnancy rates comparing the long GnRHa down-regulation protocol with the GnRH antagonist protocol, although the trend favored the long GnRHa down-regulation protocol [35]. Later, Sunkara et al. (2014) in Bologna POR patients reported that the long GnRH agonist protocol, albeit non-significantly, increased the number of mature oocytes by one oocyte compared to the GnRH antagonist protocol [36]. Moreover the cancellation rate was significantly lower for the long protocol [36]. The fact that the long GnRHa down-regulation protocol results in more oocytes as compared to the GnRH antagonist protocol is also commonly seen in expected normal responder patients. The reason is most certainly the follicular synchronization obtained after down-regulation, which for the expected POR is of utmost importance as these patients usually have increased late luteal FSH levels during their natural cycle, promoting early recruitment of the leading follicle. As one more oocyte increases the live birth rate by 5% [26, 27], the long GnRH agonist down-regulation protocol should be first line treatment for expected POR, unless a double stimulation [37] is planned for oocyte/embryo accumulation and subsequent frozen thaw embryo transfer.

### Stimulation with gonadotropins

Current evidence supports a maximum daily dose of 300 IU of rFSH in the expected POR patient as higher doses do not increase neither the clinical pregnancy rate nor the live birth rate [38, 39]. Some authors previously raised concern that stimulation per se would increase embryonic aneuploidy rates, suggesting that natural cycle IVF might be an option for the POR patient [40]. However, evidence from young oocyte donors does not support this concern [41]. Moreover, natural cycle IVF results in extremely low live birth rates in the POR patient. Thus, Polyzos et al. (2012) reported a live birth rate per cycle of 2.6% and a cumulative live birth rate of only 7% after six natural IVF cycles in Bologna POR patients, and these low live birth rates after natural cycle IVF were subsequently corroborated by others [42, 43]. In contrast, one stimulated cycle, using a daily dose of 300 IU of rFSH in the Bologna POR patient resulted in a live birth rate of 11% [18]. Thus, stimulation rather than natural cycle should be the preferred first line treatment.

## Adjuvant therapy

### Growth hormone

Growth hormone (GH) has been investigated in clinical trials as a biologically plausible add-on due to stimulation of insulin-like growth factor 1 (IGF-1) which, in animal studies, has been shown to have synergistic effects with FSH on follicular development [44]. A meta-analysis from 2010, based on four small studies with a total of 165 non-Bologna POR patients, found that the OR of live birth was 5.39 (95%CI, 1.89–15.35) in favor of GH compared to standard treatment [45]. Meanwhile, in 2016, a relatively large RCT in Bologna criteria PORs investigated the effect of adding GH (7.5 IU) from stimulation day six demonstrating no significant effect on the reproductive outcome despite significantly more oocytes retrieved in the GH group (7.58, SD 1.40) compared to the control group (4.90, SD 1.78) [46]. Although patients fulfilled the Bologna criteria at inclusion and inter-cycle variation could be blamed, the high mean number of oocytes retrieved in both groups questions the pre-stimulation ovarian reserve of the patients. In contrast, a recent retrospective analysis evaluating clinical management during 7 years of first fresh embryo transfer cycle in poor prognosis patients, i.e. patients with marked fragmentation (> 50%), implantation failure or Bologna POR, reported that GH supplementation initiated in the cycle preceding stimulation and oocyte retrieval increased the LBR although, the effect was more pronounced in the younger patient [47]. Taken together, although physiological evidence supports a clinical application of GH in POR, the equivocal results and the relatively few and small RCTs conducted emphasize the need for further studies regarding the use of adjuvant GH.

### Androgens

Another adjuvant which has been considered for the expected POR patient is androgen pretreatment. The biological evidence from a primate model is that androgens induce FSH receptors on granulosa cells [48], which in turn increases the recruitability and growth of preantral and antral follicles, including the aforementioned IGF-1 system [49, 50]. In 2012, two independent meta-analyses reported a significant positive effect of transdermal testosterone on the LBR of POR patients [51, 52]. However, this evidence should be taken with caution as only a total of 82 patients and 113 patients were included in the intervention arm of the respective meta-analyses, which again included studies performed prior to the Bologna criteria. In another meta-analysis of four RCT’s and 2 observational studies including a total of 528 patients, Zhang et al. *(2016)* reported that long-term DHEA treatment, the precursor of testosterone, had a significant positive effect on the LBR of POR patients compared to controls (RR 1.87, 95%CI, 1.22–2.88) [53]. Although scientific evidence seems to support the use of androgen pre-treatment in POR, a recent commentary argued that the androgen chapter needs further study before recommendations can be made [54]. Especially, the dosage and the timing of pre-treatment needs to be further elucidated hence; this fact urged an international clinical research group to design the so-called T-TRANSPORT TRIAL for Bologna POR patients (Clinicaltrial.gov identifier NCT02418572), evaluating pre-treatment exceeding 60 days, and using a daily dose of 5.5 mg transdermal testosterone. This study which will include the largest sample size until now of Bologna POR patients uses androgen pre-treatment in a daily physiological dose and for an extended time compared to previous trials, taking the time needed for folliculogenesis into account.

### LH supplementation

The last adjuvant for POR considered in this review is LH-activity supplementation. The physiological rationale for LH supplementation is primarily based on the “two cell two gonadotropin” concept [55, 56], in which LH supplementation stimulates the conversion of cholesterol into androgens in the theca cell, thus, increasing endogenous intra-ovarian androgen production and follicular growth. On one hand, androgens (i) stimulate FSH receptor expression in granulosa cells [48] (ii) act synergistically with IGF1 [57] and increase recruitability of pre-antral and antral follicles [58]. On the other hand, LH binding to granulosa cell LH receptors –expressed on mid-follicular phase onwards- sustains FSH-dependent granulosa activities, including aromatase induction and growth factors release, and regulates final follicle/oocyte maturation [59].

To study the possible clinical effect of rLH supplementation Lehert et al., (2014) published a meta-analysis based on 6443 cycles in normal and POR patients (non-Bologna criteria) supplemented or not with rLH [60]. While rLH supplementation improved clinical pregnancy rates by 9% (NS) in the overall population, the effect was more pronounced in PORs with RR of 1.30 (95% CI, 1.01–1.67). Recently Humaidan et al. (2017) published the results of the largest RCT in patients aligned with the Bologna criteria (ESPART trial). In this trial, a total of 939 patients were randomized to either a fixed daily dose of either 300 IU r-hFSH plus 150 IU r-hLH or r-hFSH 300 IU alone [18]. The results indicated no significant differences between groups regarding LBR. However, a post-hoc analysis stratifying patients into mild, moderate or severe POR observed that the moderate and severe PORs significantly benefitted from rLH supplementation in terms of a higher LBR and a lower total pregnancy loss [18].

## Current limitations in expected POR management

POR remains a phenotype with multiple underlying causes which future iCOS might target more adequately. In this review, we discussed recent advances in POR starting with classification and moving to current best practice iCOS, and the perspectives of adjuvants which might change the prognosis of PORs. The POSEIDON criteria take into account age as a proxy for the aneuploidy rate, as well as ovarian response if a previous stimulation was performed. Moreover, other causes of poor response in good ovarian reserve patients like polymorphisms of the FSRr and the LHr or the presence of variant LH β are covered by POSEIDON criteria. Thus, this new suggested classification of POR reduces the heterogeneity seen within the Bologna criteria, albeit POSEIDON classification still needs to be validated in clinical trials. At this point, however, the evidence for clinical management of expected POR is still limited and as discussed, few adjuvant treatments can be recommended outside institutional review board-approved research.

## Future handling of the expected POR patient

On a more experimental basis the future handling of the expected POR patient might include intra-ovarian androgen “priming” as described in the normal ovarian reserve patient [61], in vitro follicle activation as described for the POI patient [62], autologous mitochondrial transfer to improve the implantation potential and quality of the embryo [63], pharmacogenomics, taking the genome of the patient into consideration when designing drugs and planning a treatment; finally, and probably the most promising future treatment is the development of oocytes from stem cells of the patient [64].

## Conclusions

Until 2011 there was no clear definition of POR leading to a high degree of confusion. However, with the introduction of the Bologna criteria it became apparent that even this classification model described a very heterogenous group of patients with highly different success rates after ART. This lead to the recent development of the suggested POSEIDON criteria for POR, stratifying patients into more homogenous sub-groups, and importantly, giving recommendations for clinical handling. Treatment of the expected POR patient demands an individualized approach including all steps of ART, including the choice of GnRH analogue, gonadotropin type and dose, ovulation trigger, and the possible use of adjuvant therapies. Although, handling the expected POR patient still remains a therapeutic challenge, future perspectives suggest that there might be “light at the end of the tunnel”.

## Abbreviations

AFC: Antral follicle count
AMH: Anti-Mullerian hormone
ART: Assisted reproductive technology
ESHRE: European society of Human reproduction and embryology
FORT: Follicle output rate
GH: Growth hormone
iCOS: Individualized controlled ovarian stimulation
IU: International Units
OR: Odds ratio
POR: Poor ovarian response
POSEIDON: Patient oriented strategies encompassing individualized oocyte number
RCT: Randomized controlled trials
ROC: Receiver operating characteristic
RR: Relative risk
SD: Standard deviation
